# 2,4,5,6-Tetra­methyl-3-phenyl­sulfinyl-1-benzofuran

**DOI:** 10.1107/S1600536811018952

**Published:** 2011-05-25

**Authors:** Pil Ja Seo, Hong Dae Choi, Byeng Wha Son, Uk Lee

**Affiliations:** a Department of Chemistry, Dongeui University, San 24 Kaya-dong Busanjin-gu, Busan 614-714, Republic of Korea; b Department of Chemistry, Pukyong National University, 599-1 Daeyeon 3-dong, Nam-gu, Busan 608-737, Republic of Korea

## Abstract

In the title compound, C_18_H_18_O_2_S, the phenyl ring makes a dihedral angle of 87.47 (6)° with the mean plane of the benzofuran fragment. In the crystal, mol­ecules are linked by weak inter­molecular C—H⋯O hydrogen bonds and C—H⋯π inter­actions.

## Related literature

For the biological activity of benzofuran compounds, see: Aslam *et al.* (2009 ▶); Galal *et al.* (2009 ▶); Khan *et al.* (2005 ▶). For natural products with benzofuran rings, see: Akgul & Anil (2003 ▶); Soekamto *et al.* (2003 ▶). For structural studies of related 2-methyl-3-phenyl­sulfinyl-1-benzofuran derivatives, see: Choi *et al.* (2008**a* ▶,*b* ▶,c* ▶).
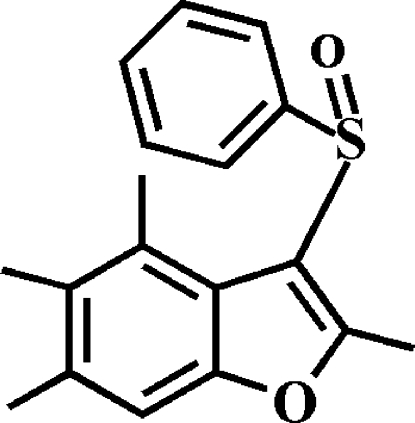


## Experimental

### 

#### Crystal data

C_18_H_18_O_2_S
*M* *_r_* = 298.38
Monoclinic, 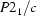

*a* = 14.2611 (5) Å
*b* = 6.0661 (2) Å
*c* = 17.2436 (7) Å
β = 99.740 (2)°
*V* = 1470.23 (9) Å^3^
*Z* = 4
Mo *K*α radiation
μ = 0.22 mm^−1^
*T* = 173 K
0.29 × 0.20 × 0.14 mm

#### Data collection

Bruker SMART APEXII CCD diffractometer
Absorption correction: multi-scan (*SADABS*; Bruker, 2009 ▶) *T*_min_ = 0.622, *T*_max_ = 0.746
25036 measured reflections
3397 independent reflections
2888 reflections with *I* > 2σ(*I*)
*R*_int_ = 0.050

#### Refinement

*R*[*F* ^2^ > 2σ(*F* ^2^)] = 0.058
*wR*(*F* ^2^) = 0.155
*S* = 1.04
3397 reflections
188 parameters
H-atom parameters constrained
Δρ_max_ = 1.20 e Å^−3^
Δρ_min_ = −0.56 e Å^−3^

### 

Data collection: *APEX2* (Bruker, 2009 ▶); cell refinement: *SAINT* (Bruker, 2009 ▶); data reduction: *SAINT*; program(s) used to solve structure: *SHELXS97* (Sheldrick, 2008 ▶); program(s) used to refine structure: *SHELXL97* (Sheldrick, 2008 ▶); molecular graphics: *ORTEP-3* (Farrugia, 1997 ▶) and *DIAMOND* (Brandenburg, 1998 ▶); software used to prepare material for publication: *SHELXL97*.

## Supplementary Material

Crystal structure: contains datablocks global, I. DOI: 10.1107/S1600536811018952/jj2090sup1.cif

Structure factors: contains datablocks I. DOI: 10.1107/S1600536811018952/jj2090Isup2.hkl

Supplementary material file. DOI: 10.1107/S1600536811018952/jj2090Isup3.cml

Additional supplementary materials:  crystallographic information; 3D view; checkCIF report

## Figures and Tables

**Table 1:** Hydrogen-bond geometry (Å, °) *Cg* is the centroid of the C2–C7 benzene ring.

*D*—H⋯*A*	*D*—H	H⋯*A*	*D*⋯*A*	*D*—H⋯*A*
C6—H6⋯O2^i^	0.95	2.35	3.286 (3)	169
C10—H10*A*⋯O2^ii^	0.98	2.56	3.517 (3)	167
C12—H12*C*⋯*Cg*^iii^	0.98	2.66	3.482 (3)	142

Symmetry codes: (i) 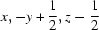; (ii) 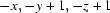; (iii) 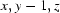.

**Fig. 1.**
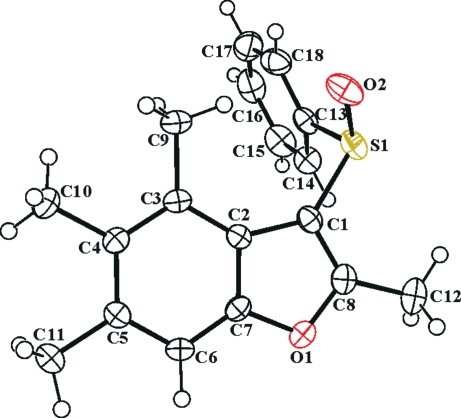
The molecular structure of the title compound with the atom numbering scheme. Displacement ellipsoids are drawn at the 50% probability level. H atoms are presented as a small spheres of arbitrary radius.

**Fig. 2.**
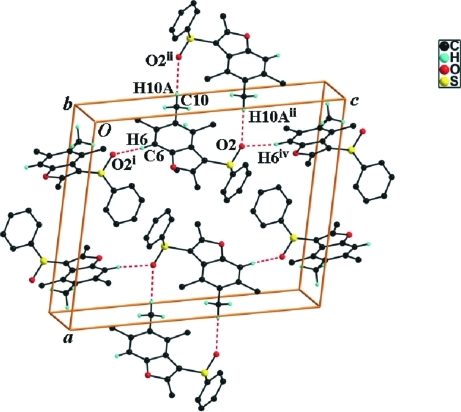
A view of the weak C—H···O interactions (dotted lines) in the crystal packing of the title compound. [Symmetry codes: (i) x, - y + 1/2 , z - 1/2; (ii) - x, - y + 1, - z + 1; (iv) x, - y + 1/2, z + 1/2.]

**Fig. 3.**
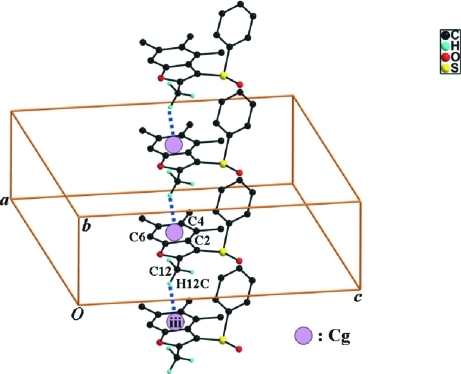
A view of the weak C—H···Cg π-ring intermolecular interactions (dotted lines) in the crystal packing of the title compound. [Symmetry codes: (iii) x, y - 1, z.]

## supplementary crystallographic information

### Comment

Recently, a series of compounds containing the benzofuran ring system have been shown to exhibit significant pharmacological properties such as antibacterial, antifungal, antitumor, antiviral, and antimicrobial activities (Aslam *et al.* , 2009, Galal *et al.* , 2009, Khan *et al.* , 2005). These types of compounds occur in a wide range of natural products (Akgul & Anil, 2003; Soekamto *et al.* , 2003). As a part of our study of the substituent effect on the solid state structures of 2-methyl-3-phenylsulfinyl-1-benzofuran analogues (Choi *et al.*, 2008**a*,*b*,c*), we report hrerin the crystal structure of the title compound, C_18_H_18_O_2_S.

In the title compound (Fig. 1), the benzofuran unit is essentially planar, with a mean deviation of 0.017 (1) Å from the least-squares plane defined by the nine constituent atoms. The phenyl ring makes a dihedral angle of 87.47 (6)° with the mean plane of the benzofuran fragment. Crystal packing is stabilized by weak intermolecular C—H···O hydrogen bonds (Table 1 & Fig. 2) and weak C—H···Cg  π–ring interactions (Table 1 & Fig 3; Cg is the centroid of the C2–C7 benzene ring).

### Experimental

77% 3-chloroperoxybenzoic acid (247 mg, 1.1 mmol) was added in small portions to a stirred solution of 2,4,5,6-tetramethyl-3-phenylsulfanyl-1-benzofuran (310 mg, 1.1 mmol) in dichloromethane (40 mL) at 273 K. After being stirred at room temperature for 4h, the mixture was washed with saturated sodium bicarbonate solution and the organic layer was separated, dried over magnesium sulfate, filtered and concentrated at reduced pressure. The residue was purified by column chromatography (hexane-ethyl acetate, 2:1 v/v) to afford the title compound as a colorless solid [yield 74%, m.p. 438–439 K; *R*_f_ = 0.51 (hexane–ethyl acetate, 2:1 v/v)]. Single crystals suitable for X-ray diffraction were prepared by slow evaporation of a solution of the title compound in ethyl acetate at room temperature.

### Refinement

All H atoms were positioned geometrically and refined using a riding model, with C—H = 0.95 Å for aryl and 0.99 Å for methyl H atoms. *U*_iso_(H)  = 1.2*U*_eq_(C)  for aryl and 1.5*U*_eq_(C) for methyl H atoms.

### Crystal data

**Table:**

C_18_H_18_O_2_S	*F*(000) = 632
*M**_r_* = 298.38	*D*_x_ = 1.348 Mg m^−^^3^
Monoclinic, *P*2_1_/*c*	Mo *K*α radiation, λ = 0.71073 Å
Hall symbol: -P 2ybc	Cell parameters from 7044 reflections
*a* = 14.2611 (5) Å	θ = 2.4–27.5°
*b* = 6.0661 (2) Å	µ = 0.22 mm^−^^1^
*c* = 17.2436 (7) Å	*T* = 173 K
β = 99.740 (2)°	Block, colourless
*V* = 1470.23 (9) Å^3^	0.29 × 0.20 × 0.14 mm
*Z* = 4	

### Data collection

**Table:**

Bruker SMART APEXII CCD diffractometer	3397 independent reflections
Radiation source: rotating anode	2888 reflections with *I* > 2σ(*I*)
graphite multilayer	*R*_int_ = 0.050
Detector resolution: 10.0 pixels mm^-1^	θ_max_ = 27.6°, θ_min_ = 1.5°
φ and ω scans	*h* = −18→17
Absorption correction: multi-scan (*SADABS*; Bruker, 2009)	*k* = −7→7
*T*_min_ = 0.622, *T*_max_ = 0.746	*l* = −22→22
25036 measured reflections	

### Refinement

**Table:**

Refinement on *F*^2^	Primary atom site location: structure-invariant direct methods
Least-squares matrix: full	Secondary atom site location: difference Fourier map
*R*[*F*^2^ > 2σ(*F*^2^)] = 0.058	Hydrogen site location: difference Fourier map
*wR*(*F*^2^) = 0.155	H-atom parameters constrained
*S* = 1.04	*w* = 1/[σ^2^(*F*_o_^2^) + (0.0691*P*)^2^ + 1.9351*P*] where *P* = (*F*_o_^2^ + 2*F*_c_^2^)/3
3397 reflections	(Δ/σ)_max_ = 0.001
188 parameters	Δρ_max_ = 1.20 e Å^−^^3^
0 restraints	Δρ_min_ = −0.56 e Å^−^^3^

### Special details

**Table:**

Geometry. All esds (except the esd in the dihedral angle between two l.s. planes) are estimated using the full covariance matrix. The cell esds are taken into account individually in the estimation of esds in distances, angles and torsion angles; correlations between esds in cell parameters are only used when they are defined by crystal symmetry. An approximate (isotropic) treatment of cell esds is used for estimating esds involving l.s. planes.
Refinement. Refinement of F^2^ against ALL reflections. The weighted R-factor wR and goodness of fit S are based on F^2^, conventional R-factors R are based on F, with F set to zero for negative F^2^. The threshold expression of F^2^ > 2sigma(F^2^) is used only for calculating R-factors(gt) etc. and is not relevant to the choice of reflections for refinement. R-factors based on F^2^ are statistically about twice as large as those based on F, and R- factors based on ALL data will be even larger.

### Fractional atomic coordinates and isotropic or equivalent isotropic displacement parameters (Å^2^)

**Table:**

	*x*	*y*	*z*	*U*_iso_*/*U*_eq_	
S1	0.28526 (4)	0.15501 (10)	0.58076 (4)	0.03155 (19)	
O1	0.27241 (11)	0.1859 (3)	0.35463 (10)	0.0271 (4)	
O2	0.19888 (14)	0.1527 (3)	0.61794 (11)	0.0420 (5)	
C1	0.25752 (15)	0.2287 (4)	0.48136 (13)	0.0251 (5)	
C2	0.20419 (15)	0.4077 (3)	0.43714 (12)	0.0219 (4)	
C3	0.14688 (15)	0.5842 (4)	0.45266 (12)	0.0225 (4)	
C4	0.10995 (15)	0.7234 (4)	0.39007 (13)	0.0233 (4)	
C5	0.12764 (15)	0.6842 (4)	0.31306 (13)	0.0239 (4)	
C6	0.18162 (15)	0.5044 (4)	0.29734 (13)	0.0245 (5)	
H6	0.1930	0.4739	0.2457	0.029*	
C7	0.21775 (15)	0.3723 (3)	0.36019 (13)	0.0230 (4)	
C8	0.29453 (16)	0.1033 (4)	0.42877 (14)	0.0275 (5)	
C9	0.12387 (18)	0.6209 (4)	0.53373 (14)	0.0302 (5)	
H9A	0.1379	0.4866	0.5651	0.045*	
H9B	0.0563	0.6572	0.5297	0.045*	
H9C	0.1624	0.7428	0.5592	0.045*	
C10	0.04905 (17)	0.9172 (4)	0.40466 (15)	0.0317 (5)	
H10A	−0.0180	0.8728	0.3956	0.048*	
H10B	0.0581	1.0374	0.3687	0.048*	
H10C	0.0675	0.9672	0.4591	0.048*	
C11	0.08671 (18)	0.8332 (4)	0.24575 (14)	0.0320 (5)	
H11A	0.1034	0.7760	0.1967	0.048*	
H11B	0.1128	0.9819	0.2555	0.048*	
H11C	0.0173	0.8384	0.2413	0.048*	
C12	0.35458 (14)	−0.0982 (3)	0.43559 (11)	0.0378 (6)	
H12A	0.3646	−0.1510	0.4901	0.057*	
H12B	0.4162	−0.0636	0.4205	0.057*	
H12C	0.3226	−0.2129	0.4007	0.057*	
C13	0.35299 (11)	0.3951 (3)	0.61796 (9)	0.0275 (5)	
C14	0.43833 (10)	0.4325 (2)	0.59220 (10)	0.0327 (5)	
H14	0.4567	0.3431	0.5521	0.039*	
C15	0.49648 (19)	0.6014 (5)	0.62557 (17)	0.0423 (7)	
H15	0.5556	0.6278	0.6088	0.051*	
C16	0.4686 (2)	0.7322 (5)	0.68343 (17)	0.0456 (7)	
H16	0.5083	0.8495	0.7059	0.055*	
C17	0.3838 (2)	0.6929 (5)	0.70828 (17)	0.0452 (7)	
H17	0.3647	0.7840	0.7476	0.054*	
C18	0.32583 (18)	0.5214 (5)	0.67638 (15)	0.0370 (6)	
H18	0.2679	0.4914	0.6947	0.044*	

### Atomic displacement parameters (Å^2^)

**Table:**

	*U*^11^	*U*^22^	*U*^33^	*U*^12^	*U*^13^	*U*^23^
S1	0.0337 (3)	0.0300 (3)	0.0297 (3)	−0.0058 (2)	0.0016 (2)	0.0081 (2)
O1	0.0258 (8)	0.0250 (8)	0.0309 (9)	0.0009 (6)	0.0059 (7)	−0.0045 (6)
O2	0.0397 (10)	0.0523 (12)	0.0347 (10)	−0.0122 (9)	0.0086 (8)	0.0101 (8)
C1	0.0245 (11)	0.0244 (10)	0.0256 (11)	−0.0046 (8)	0.0017 (8)	0.0033 (8)
C2	0.0219 (10)	0.0221 (10)	0.0213 (10)	−0.0044 (8)	0.0024 (8)	0.0005 (8)
C3	0.0207 (10)	0.0256 (10)	0.0217 (10)	−0.0055 (8)	0.0052 (8)	−0.0042 (8)
C4	0.0194 (10)	0.0244 (10)	0.0269 (11)	−0.0021 (8)	0.0062 (8)	−0.0016 (8)
C5	0.0204 (10)	0.0266 (10)	0.0238 (11)	−0.0028 (8)	0.0017 (8)	0.0010 (8)
C6	0.0252 (11)	0.0294 (11)	0.0194 (10)	−0.0028 (8)	0.0049 (8)	−0.0032 (8)
C7	0.0200 (10)	0.0226 (10)	0.0264 (11)	−0.0017 (8)	0.0037 (8)	−0.0051 (8)
C8	0.0225 (11)	0.0236 (10)	0.0355 (12)	−0.0035 (8)	0.0025 (9)	0.0002 (9)
C9	0.0330 (12)	0.0349 (12)	0.0245 (11)	−0.0019 (9)	0.0102 (9)	−0.0039 (9)
C10	0.0283 (12)	0.0305 (12)	0.0373 (13)	0.0050 (9)	0.0085 (10)	−0.0017 (10)
C11	0.0336 (13)	0.0339 (12)	0.0274 (12)	0.0009 (10)	0.0017 (10)	0.0054 (10)
C12	0.0329 (13)	0.0263 (12)	0.0535 (16)	0.0031 (10)	0.0051 (12)	0.0010 (11)
C13	0.0255 (11)	0.0303 (11)	0.0249 (11)	−0.0017 (9)	−0.0013 (9)	0.0069 (9)
C14	0.0273 (12)	0.0362 (13)	0.0342 (13)	−0.0013 (10)	0.0041 (10)	0.0017 (10)
C15	0.0318 (14)	0.0471 (16)	0.0460 (16)	−0.0116 (11)	0.0011 (12)	0.0062 (12)
C16	0.0509 (17)	0.0362 (14)	0.0429 (16)	−0.0085 (12)	−0.0119 (13)	0.0010 (12)
C17	0.0554 (18)	0.0440 (15)	0.0322 (14)	0.0096 (13)	−0.0046 (12)	−0.0070 (12)
C18	0.0327 (13)	0.0504 (15)	0.0271 (12)	0.0049 (11)	0.0024 (10)	0.0034 (11)

### Geometric parameters (Å, °)

**Table:**

S1—O2	1.482 (2)	C10—H10A	0.9800
S1—C1	1.751 (2)	C10—H10B	0.9800
S1—C13	1.8046 (18)	C10—H10C	0.9800
O1—C8	1.359 (3)	C11—H11A	0.9800
O1—C7	1.386 (3)	C11—H11B	0.9800
C1—C8	1.357 (3)	C11—H11C	0.9800
C1—C2	1.463 (3)	C12—H12A	0.9800
C2—C7	1.390 (3)	C12—H12B	0.9800
C2—C3	1.400 (3)	C12—H12C	0.9800
C3—C4	1.401 (3)	C13—C18	1.372 (3)
C3—C9	1.506 (3)	C13—C14	1.3830
C4—C5	1.413 (3)	C14—C15	1.381 (3)
C4—C10	1.508 (3)	C14—H14	0.9500
C5—C6	1.388 (3)	C15—C16	1.385 (4)
C5—C11	1.508 (3)	C15—H15	0.9500
C6—C7	1.376 (3)	C16—C17	1.370 (5)
C6—H6	0.9500	C16—H16	0.9500
C8—C12	1.486 (3)	C17—C18	1.384 (4)
C9—H9A	0.9800	C17—H17	0.9500
C9—H9B	0.9800	C18—H18	0.9500
C9—H9C	0.9800		
			
O2—S1—C1	111.05 (11)	C4—C10—H10B	109.5
O2—S1—C13	106.72 (10)	H10A—C10—H10B	109.5
C1—S1—C13	99.25 (9)	C4—C10—H10C	109.5
C8—O1—C7	106.22 (17)	H10A—C10—H10C	109.5
C8—C1—C2	107.1 (2)	H10B—C10—H10C	109.5
C8—C1—S1	117.52 (18)	C5—C11—H11A	109.5
C2—C1—S1	135.31 (18)	C5—C11—H11B	109.5
C7—C2—C3	118.7 (2)	H11A—C11—H11B	109.5
C7—C2—C1	103.83 (19)	C5—C11—H11C	109.5
C3—C2—C1	137.4 (2)	H11A—C11—H11C	109.5
C2—C3—C4	117.98 (19)	H11B—C11—H11C	109.5
C2—C3—C9	121.1 (2)	C8—C12—H12A	109.5
C4—C3—C9	120.9 (2)	C8—C12—H12B	109.5
C3—C4—C5	121.3 (2)	H12A—C12—H12B	109.5
C3—C4—C10	119.6 (2)	C8—C12—H12C	109.5
C5—C4—C10	119.2 (2)	H12A—C12—H12C	109.5
C6—C5—C4	120.6 (2)	H12B—C12—H12C	109.5
C6—C5—C11	118.4 (2)	C18—C13—C14	121.16 (15)
C4—C5—C11	121.0 (2)	C18—C13—S1	120.72 (16)
C7—C6—C5	116.7 (2)	C14—C13—S1	117.72 (6)
C7—C6—H6	121.6	C15—C14—C13	119.12 (16)
C5—C6—H6	121.6	C15—C14—H14	120.4
C6—C7—O1	124.1 (2)	C13—C14—H14	120.4
C6—C7—C2	124.6 (2)	C14—C15—C16	120.0 (2)
O1—C7—C2	111.25 (19)	C14—C15—H15	120.0
C1—C8—O1	111.6 (2)	C16—C15—H15	120.0
C1—C8—C12	133.8 (2)	C17—C16—C15	120.1 (3)
O1—C8—C12	114.61 (19)	C17—C16—H16	119.9
C3—C9—H9A	109.5	C15—C16—H16	119.9
C3—C9—H9B	109.5	C16—C17—C18	120.4 (3)
H9A—C9—H9B	109.5	C16—C17—H17	119.8
C3—C9—H9C	109.5	C18—C17—H17	119.8
H9A—C9—H9C	109.5	C13—C18—C17	119.2 (2)
H9B—C9—H9C	109.5	C13—C18—H18	120.4
C4—C10—H10A	109.5	C17—C18—H18	120.4
			
O2—S1—C1—C8	−132.93 (19)	C8—O1—C7—C6	179.6 (2)
C13—S1—C1—C8	115.04 (18)	C8—O1—C7—C2	−0.6 (2)
O2—S1—C1—C2	50.2 (3)	C3—C2—C7—C6	2.6 (3)
C13—S1—C1—C2	−61.8 (2)	C1—C2—C7—C6	−178.8 (2)
C8—C1—C2—C7	−1.7 (2)	C3—C2—C7—O1	−177.22 (18)
S1—C1—C2—C7	175.39 (19)	C1—C2—C7—O1	1.4 (2)
C8—C1—C2—C3	176.5 (2)	C2—C1—C8—O1	1.4 (2)
S1—C1—C2—C3	−6.4 (4)	S1—C1—C8—O1	−176.26 (14)
C7—C2—C3—C4	−3.2 (3)	C2—C1—C8—C12	179.9 (2)
C1—C2—C3—C4	178.8 (2)	S1—C1—C8—C12	2.2 (4)
C7—C2—C3—C9	175.6 (2)	C7—O1—C8—C1	−0.6 (2)
C1—C2—C3—C9	−2.4 (4)	C7—O1—C8—C12	−179.34 (18)
C2—C3—C4—C5	1.7 (3)	O2—S1—C13—C18	6.54 (19)
C9—C3—C4—C5	−177.1 (2)	C1—S1—C13—C18	121.94 (17)
C2—C3—C4—C10	−179.1 (2)	O2—S1—C13—C14	179.34 (9)
C9—C3—C4—C10	2.1 (3)	C1—S1—C13—C14	−65.26 (9)
C3—C4—C5—C6	0.7 (3)	C18—C13—C14—C15	−0.62 (19)
C10—C4—C5—C6	−178.5 (2)	S1—C13—C14—C15	−173.38 (18)
C3—C4—C5—C11	179.3 (2)	C13—C14—C15—C16	−0.7 (3)
C10—C4—C5—C11	0.1 (3)	C14—C15—C16—C17	0.7 (4)
C4—C5—C6—C7	−1.4 (3)	C15—C16—C17—C18	0.6 (4)
C11—C5—C6—C7	180.0 (2)	C14—C13—C18—C17	1.9 (3)
C5—C6—C7—O1	179.54 (19)	S1—C13—C18—C17	174.44 (19)
C5—C6—C7—C2	−0.3 (3)	C16—C17—C18—C13	−1.9 (4)

### Hydrogen-bond geometry (Å, °)

**Table:**

Cg is the centroid of the C2–C7 benzene ring.

**Table:**

*D*—H···*A*	*D*—H	H···*A*	*D*···*A*	*D*—H···*A*
C6—H6···O2^i^	0.95	2.35	3.286 (3)	169.
C10—H10A···O2^ii^	0.98	2.56	3.517 (3)	167.
C12—H12C···Cg^iii^	0.98	2.66	3.482 (3)	142.

Symmetry codes: (i) *x*, −*y*+1/2, *z*−1/2; (ii) −*x*, −*y*+1, −*z*+1; (iii) *x*, *y*−1, *z*.
